# The degree of toxoplasmosis and testicular histomorphometry in rats

**DOI:** 10.1038/s41598-024-78653-3

**Published:** 2024-11-13

**Authors:** Mamdouh Hegazy, Walaa Elghanam, Nora Aboulfotouh, Heba Sheta, Nora El-Tantawy

**Affiliations:** 1 Department of Medical Parasitology, Faculty of Medicine, Mansoura University, Mansoura, Egypt; 2 Department of Pathology, Faculty of Medicine, Mansoura University, Mansoura, Egypt

**Keywords:** *T. Gondii*, Histopathology, Brain, Fertility, Cyst, Wistar rat, Male, Microbiology, Zoology, Medical research

## Abstract

Toxoplasma gondii (T. gondii) ranks as the third most common parasitic parasite worldwide, and it is estimated that > 60% of the population is infected with T. gondii worldwide at some point in their lives. So. Therefore, it is highly curious that T. gondii could be a potential cause of idiopathic infertility that is incidental to male partners, who are responsible for nearly 50% of cases. Testicular histomorphometric analysis was developed to investigate fertility problems. The objective of this experimental study was to assess the impact of toxoplasmosis on spermatogenesis indicated by histomorphometric changes in rat testes and its correlation with the degree of infection in the brain. Ninety male Wistar albino rats were infected with T. gondii, and 30 male rats composed the control group. The studied parameters were investigated from the seventh week until the twelfth week postinfection by estimating the body weight, the weight of the testes, histopathological examination, and metric analysis of the testes. Each time, correlations were detected between the investigated parameters and the infection severity calculated by estimating the cyst burden in brain homogenates and brain lesion grading of stained histological sections.Our findings demonstrated a significant adverse impact of toxoplasmosis on absolute body weight, testis weight, and testis histomorphometry. The grading of brain lesions and the number of brain cysts paralleled each other. There was a reverse correlation between the gonado-somatic index and the number of brain cysts and brain lesion grade. There was a statistically significant correlation between the brain cyst count and the brain lesion grade and the indices 20 A, 20b, 250, and 200 of the testes metric analysis. Conclusion: Our results revealed that toxoplasmosis has an adverse impact on male rat spermatogenic cells, which in turn affects spermatogenesis and fertility. This impact is significantly correlated with the degree of latent infection in the brain.

**Supplementary Information:**

The online version contains supplementary material available at 10.1038/s41598-024-78653-3.

## Introduction

Approximately one-third of human and animal populations are infected with the intracellular protozoan parasite *Toxoplasma gondii (T. gondii*), which causes toxoplasmosis. It is a prevalent infection worldwide^1^. Humans can become infected by eating raw or undercooked meat that contains *T. gondii* tissue cysts or by drinking polluted water or food that contains oocytes excreted in cat feces^2^. Other methods of infection include congenital transmission from infected mothers to their fetuses, organ transplants, and blood transfusions from infected to uninfected people^3^. Furthermore, recent research has shown that *T. gondii* can spread through sexual contact in humans^4^ and Brown rats^5^. Due to its diverse range of warm-blooded animal hosts and the potential for infection in approximately one-third of the population globally, *T. gondii* is the most regarded protozoan globally^6^. The prevalence of *T. gondii* infection varied substantially worldwide^7^. For instance, in highly endemic regions such as areas of Africa, the incidence of toxoplasmosis could exceed 90% in some demographic groups, whereas in specific populations in Europe, it could be as low as 60%^3^.

During the course of the infection, the illness’s clinical symptoms remain chronic and subclinical^8^. Many infected individuals are unaware that they are infected since toxoplasmosis does not always cause pathological issues in the host, especially in individuals who have strong body immunity^9^. Given the disease’s chronic nature, high rate of transmission in humans, serious consequences, and general lack of knowledge about the disease, toxoplasmosis infections are regarded as a significant threat to public health^10^.

*gondii* becomes dormant as bradyzoites in tissue cysts, which can be found in the central nervous system, lungs, eyes, liver, kidneys or skeletal and cardiac muscles, and *T. gondii* survives in its hosts for the remainder of its life^2^. Furthermore, semen and reproductive organs have been reported to have *T. gondii* cysts. *T. gondii* DNA has been detected in the semen of male goats^11^ and rabbits^12^. In contrast, *T. gondii* cysts have been detected in the testes of male mice^13^ and in the semen and epididymis of male rats^5^. Tachyzoites have been identified in the testicles, epididymides, and semen of dogs affected by acute toxoplasmosis^14^. *T. gondii* was identified in the testes, seminal vesicles, and semen of bovine samples^15^, as well as in the pig epididymis and seminal vesicles^16^.

Up to half of all couples seeking medical care for infertility struggle to identify the exact cause of their infertility. Thus, these patients are classified as having idiopathic infertility^17^. Male factors are the exclusive cause of infertility in approximately 20% of cases and contribute to infertility in another 30–40% of cases; therefore, male factors are involved in more than 50% of cases when couples attempt to conceive^18^. Reduced sperm quality is one cause of male infertility that might be due to a decrease in seminal plasma fructose levels, which is essential for the normality of sperm motility^19^, and excess production of reactive oxygen species, which could have a detrimental effect on the quality of sperm^20^. Many studies have established associations between male genital tract damage and toxoplasmosis-related hypogonadotropic hypogonadism, testicular inflammation^21^, testicular damage^22^, and sperm dysfunction^23^ and between male genital tract damage and decreased testosterone levels^24^.

The reproductive organ of mammals, the testis, has several compartments, including the seminiferous tubules, interstitium, and other cell types. Spermatogenesis occurs in the seminiferous tubules, while androgen biosynthesis and paracrine secretion occur in the interstitium^25^. The interstitium includes Leydig cells, blood vessels, lymphatic vessels, and nerves of the testicular parenchyma^26^. A crucial indicator of spermatogenic activity that provides insight into the degree of spermatogenesis is the histological assessment of the testicular parenchyma^27,28^. A number of animal species, including humans, have undergone quantitative testicular histomorphometric evaluations^29–32^. These evaluations have been applied to studies of both physiological and pathological conditions^33,34^. Thus, to evaluate the aspects of reproductive biology, several studies have been conducted based on qualitative and quantitative assessments of the spermatogenic process, including histomorphometry^35,36^.

Overall, the current data indicate a possible association between male toxoplasmosis and infertility^24,37^. Histomorphometric examination of testes has a notable role in evaluating male reproductive ability. It could provide information on the spermatogenesis process. Thus, the present study aimed to determine whether rat testicular histological and histomorphometric changes are affected by *T. gondii* infection to assume that toxoplasmosis may impact rat fertility. In addition, these changes correlated with the degree of toxoplasmosis infection, as indicated by the number of *T. gondii* brain cysts and the degree of brain toxoplasmosis lesions.

## Subjects and methods

The study was conducted at the Medical Pathology and Parasitology departments of the Faculty of Medicine at Mansoura University, Egypt. The study was carried out according to the “Guide for the Care and Use of Laboratory Animals” distributed by the National Institutes of Laboratory Animal Resources, United States. Pentobarbital sodium anesthesia was utilized for all procedures, and every attempt was made to reduce the amount of rats used and the amount of misery that the animals encountered. The study proposal was evaluated and approved by the Institutional Review Board of the Faculty of Medicine at Mansoura University in Egypt. The study was conducted strictly under the requirements of the Institutional Council on Research Ethics and is reported in accordance with ARRIVE guidelines. All experiments were performed in accordance with relevant guidelines and regulations.

### Experimental animals

As an experimental animal model, 90 laboratory-bred adult male Wistar albino rats aged twelve to fourteen weeks were infected orally with the ME49 type II cytogenic *T. gondii* strain. Additionally, thirty additional naïve Wistar albino rats were assigned to the control group. All rats were acquired from a commercial breeder (Medical Experimental research Center MERC, Mansoura University, Mansoura, Egypt). Before infection induction, an electronic weight balance was used to weigh both the test and control group rats.

### Maintenance of the T. gondii strain and tachyzoite inoculum preparation

By using a 22-gauge blunt feeding needle to continue oral passage in Swiss Webster mice, the cystogenic ME49 *T. gondii* type II strain was constantly retained. The quantity of brain homogenate that was used was adjusted to include ten tissue cysts. The mouse brains were removed from the infected rats at least eight weeks after oral infection and homogenized in a tissue homogenizer with 1 milliliter of pH 7.2 buffered saline. At ×400 magnification, the number of cysts per 10 µl of the homogenate was measured on a hemocytometer. The suspension was then diluted to a concentration of 100 cysts/ml according to Djurkovic-Djakovic et al.^38^. Using a 22-gauge blunt feeding needle, the test group of rats was intragastrically injected with a brain homogenate volume that was adjusted to include 1 × 10^3^ cysts per rat. The same volume of sterile phosphate-buffered saline was used to infect naive control rats.

### Histologic and microscopic analysis of Toxoplasma cysts in the brain

The brain was examined for *T. gondii* brain cysts to confirm infection when *Toxoplasma*-infected rats were euthanized. Brains free of *Toxoplasma* cysts were not included. The rat brain was divided in half, dissolved in phosphate buffer solution (PBS), centrifuged at 1500 × g for 10 min, and then examined under a microscope to identify *Toxoplasma* cysts^38^. The white and gray matter of the cerebrum, together with other portions of the other half of the brain, were harvested and maintained for subsequent histological examination at pH 7.2 in formaldehyde (10% in PBS) using hematoxylin and eosin (H&E) staining^39^.

### Estimating the weight of the testes

Gonadectomy was carried out via the open castration approach, which was based on the protocol of Saba et al.^40^. The testicular weights were calculated relative to 100 g of body weight employing this formula, which was developed by Stahl et al.^41^ (mean weight of both testes in grams/final body weight before necropsy in grams) ×100 is the gonadosomatic index, or GSI.

### Testicular histomorphometric analysis

Histological and metric analyses of testicular tissue were performed to assess the process of spermatogenesis. The testes were fixed for 48 h in Bouin’s solution, which is composed of 10 milliliters of glacial acetic acid, 50 milliliters of 40% formaldehyde, and 150 milliliters of saturated picric acid. The whole testicular tissue is totally sectioned in five tissue sections and all were subjected to paraffin wax embedding, xylol deparaffinization, and ethanol dehydration at increasing concentrations. Canadian balsam-covered 5 μm thick sections were cut from five blocks per rat using microtome and stained with H&E. The examination was performed using a light Olympus binuclear microscope with an integrated, calibrated ocular micrometer.

The following spermatogenic indicators were evaluated in accordance with Dvorakova Hortova et al.^42^ to assess the impact of *T. gondii* infection on the process of spermatogenesis. (i) The number of Sertoli cells/20 seminiferous tubule cross sections is shown in Index 20 A. The number of Sertoli cells in nonserially cut sections from each of the 5 serially cut sections from each of the 5 blocks/rat was counted using twenty seminiferous tubule cross sections from randomly chosen optical fields. Blocks/rats were counted concurrently for both types of cells to obtain the mean for each cell type. (ii) Index 20. B: The quantity of spherical spermatids found in twenty seminiferous tubule cross sections, such as Sertoli cells, was determined by counting the spherical spermatids in twenty nonserially cut sections from each of the five blocks/rat, which were obtained from randomly selected optical fields. Therefore, the means for each of the two types of cells were determined by counting them concurrently. (iii) Index 50 (fifty seminiferous tubule minor diameters): Five blocks per rat were used to measure the minor diameters of fifty seminiferous tubules from optical fields in nonserthally cut sections selected randomly. The center of the tubule cross section was measured for two diameters at right angles, and the mean was computed. (iv) The primary leptotene spermatocyte count/250 Sertoli cells was measured by the Index 250. Optical fields in nonserially cut sections were used to determine the number of leptotene spermatocytes/250 Sertoli cells in 5 randomly selected blocks per rat. (v) The degree of seminiferous tubule degeneration in *Toxoplasma*-infected rats (degree index; index 200): Spermatogenic cells displayed vacuolar degeneration in degenerated seminiferous tubules. Inside these tubules, there was no sperm. The grade of degeneration in this study was estimated using the proportion of degenerated tubules among 200 seminiferous tubule cross sections from randomly chosen optical fields in nonserially cut sections in each of the five blocks per rat. (Percentage of degenerated tubules = (number of degenerated tubules/200) X100).

Grade 0 indicates no lesion, Grade I indicates approximately 1 to 10% minor tubular degeneration, Grade II indicates approximately 10 to 20% mild tubular degeneration, Grade III indicates approximately 20 to 30% moderate tubular degeneration, and Grade IV indicates more than 30% severe degeneration of the tubules. The proportion of degenerating tubules determines the degree of degeneration.

### Assessment of the degree of infection

At each time point of the experiment, correlations between body weight, testis weight, testis histomorphometry and the degree of infection were determined as follows:

### Brain cyst count

At necropsy, brains were extracted, and 1/2 of the brain from each rat was used for histological study. The other 1/2 was homogenized in 1 ml of PBS (pH 7.2). The brain cyst load was calculated by counting the number of cysts in 10 µl of brain homogenate (unstained or Giemsa-stained). According to Kaňková et al.^43^, the brain cyst load was calculated as the number of brain cysts in 10 µl of homogenate ×100 × 2.

### Grading of the brain lesions

One hemisphere of the brain from each rat was fixed in 5% formalin for 48 h. With five blocks per rat, the tissue was routinely prepared for paraffin sectioning at a thickness of 5 μm and then stained with H&E as mentioned before. Lesion scoring was performed in two randomly selected microscopic fields from two nonserially cut sections cut from the 5 blocks for each rat. The brain lesions in each animal were graded according to^44,45^, where Grade 0 indicates no lesion, Grade I indicates a single minimum lesion confined to localized perivascular cuffing and slight mononuclear cell infiltration in the meninges, Grade II indicates extensive minimum lesions or mild lesions, which include perivascular cuffing, meningitis and local glial cell infiltration, Grade III indicates a single moderate lesion with perivascular cuffing, meningitis and focal necrosis with some macrophage infiltration, and Grade IV indicates extensive moderate lesions or severe lesions, including perivascular cuffing, meningitis and focal extensive necrosis. The median lesion grade of each animal in each group was calculated.

### Statistical analysis

Version 20.0 of the SPSS software program (SPSS, Inc., Chicago, Illinois, USA) was used to stratify, tabulate, and statistically analyze the data. The data normality tested using Kolmogorov–Smirnov test and the data was normally distributed. Differences and associations between qualitative variables were analyzed using the chi-square test. Student’s t test and ANOVA were applied to test parametric quantitative data, which are reported as the means. The medians were used to express non-parametric quantitative data, which were then analyzed using the Kruskal‒Wallis and Mann‒Whitney U tests. Dunnett’s or Tukey’s post hoc tests were used to compare groups across groups. Pearson’s and Spearman’s tests were used to examine correlations between the variables. *P* values less than 0.05 were considered to indicate statistical significance.

## Results

### Impact of latent T. gondii infection on the absolute body weight, testis weight and gonadosomatic index

Throughout the experimental observation period, the body weight of the control rats gradually increased, although this increase was not statistically significant (*P* = .628). Conversely, post hoc testing revealed that *Toxoplasma*-infected rats showed a substantial (*P*˂0.001) progressive decrease in body weight throughout the experimental observation period, with a peak loss occurring during the ninth and eleventh weeks after infection. In terms of testicular weight, post hoc testing revealed a highly significant, gradual decline in the absolute testicular weights of the *T. gondii*-infected rats and a marginal increase in the weights of the testes of the control rats, peaking in the eleventh and twelfth weeks following infection. In this study, control rats exhibited a non significant gradual increase in the gonadosomatic index, while *Toxoplasma-*infected rats displayed a significant gradual decrease that was more prominent in the eleventh and twelfth weeks of observation, as shown in Table 1.

**Table 1:** Mean relative weights of testes (gonadosomatic indices) of rats infected with *T. Gondii* and control rats at necropsy.

Infection duration in weeks	Control rats			Rats infected with T.gondii			Significance*	
	*n*	GSI	SD	*n*	GSI	SD	*n*	GSI
**7**	5	1.04	±0.012	15	0.96	**7**	5	1.04
**8**	5	1.039	±0.009	15	.931^**a**^	**8**	5	1.039
**9**	5	1.034	±0.014	15	0.909 ^**a**^	**9**	5	1.034
**10**	5	1.04	±0.012	15	0.890 ^**ab**^	**10**	5	1.04
**11**	5	1.04	±0.010	15	0.825 ^**abcd**^	**11**	5	1.04
**12**	5	1.042	±0.010	15	0.780 ^**abcd**^	**12**	5	1.042
Significance**	**f= 2.22**			**f=161.36 **				
	** P =.06**			**P < .001**				

GSI: Gonadosomatic index (g/100 g body weight).

n: number of rats necropstratized at each time point.

*: Student’s t test (*p* < .05), ** One-way ANOVA test and Tukey’s post hoc test (significant if *p* < .05), a: Significance vs. the 7th week, b: Significance vs. the 8th week, c: Significance vs. the 9th week, d: Significance vs. the 10th week.

### Histomorphometric analysis

**Fig. 1:**
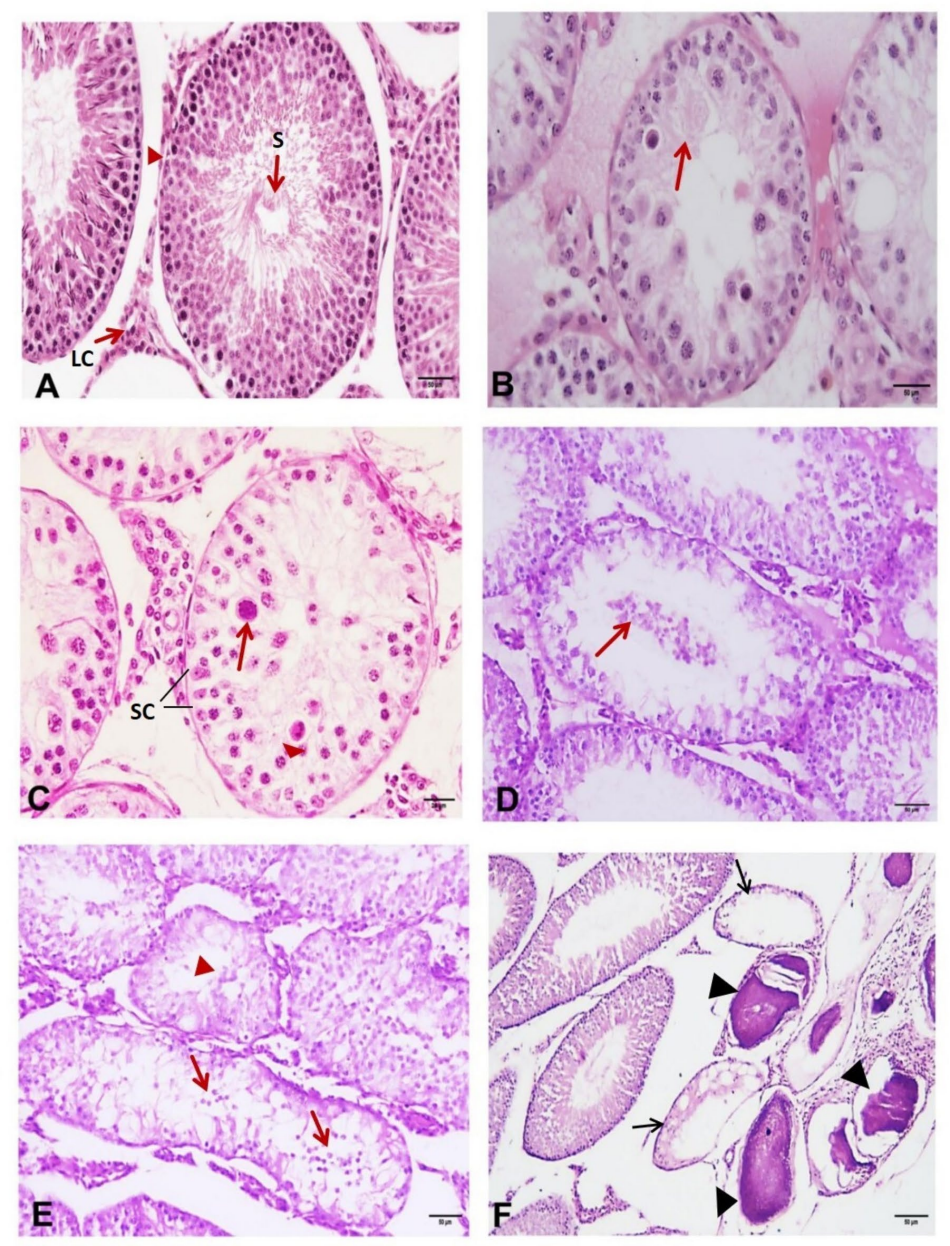
Histological analysis of the testes of the rats (H&E, ×200). (**A**) A section of a control rat testis demonstrating the normal structure of the seminiferous tubules; Arrow head: normal seminiferous tubule full with spermatogenic cells and sperm; LC: Leydig cells; S indicates spermatozoa; Section from the testis of Toxoplasma-infected rat showing (**B**) vacuolar degeneration (arrow) and necrosis of spermatogenic cells (H&E, ×200); (**C**) Toxoplasma cyst (arrow), degeneration of spermatogenic cells with apoptotic changes (arrow head) and absent spermatids (H&E, ×400); SC: Sertoli cells; (**D**) detached germ cells (arrow) in the lumen of degenerated seminiferous tubules (H&E, ×200); (**E**) irregularly outlined seminiferous tubules with degenerated spermatogenic cells, detached germ cells (arrow) in the lumen and no sperm (arrow head) (H&E, ×200); (**F**) degenerated seminiferous tubules with absence of germ cells (arrow) and dystrophic calcification (arrow heads) (H&E, ×200).

In testicular histological sections from the control rats, the seminiferous tubules appeared adjacent to each other and had a normal shape and regular outline. The average thickness of the epithelial lining was normal. The tubules are lined with germ cells arranged in concentric layers. Spermatogenesis was completed, and all stages of the spermatogenic cycle could be observed. Typical triangular or ovoid Sertoli cell nuclei displaying obvious nucleoli were observed in the basal compartment. The lumens of the seminiferous tubules were filled with spermatozoa. The interstitial tissue in the intertubular spaces consisted of a network of loose tissue with flattened fibroblasts, mast cells, macrophages, capillaries, venules, lymphatics and Leydig cells (Fig. 1A). Testicular sections from a group of rats infected with *Toxoplasma* were examined and stained with H&E for histological analysis. A variety of cell injuries, ranging from necrosis and apoptosis to vacuolar degeneration, were observed in the cells lining the seminiferous tubules.

**Fig. 2:**
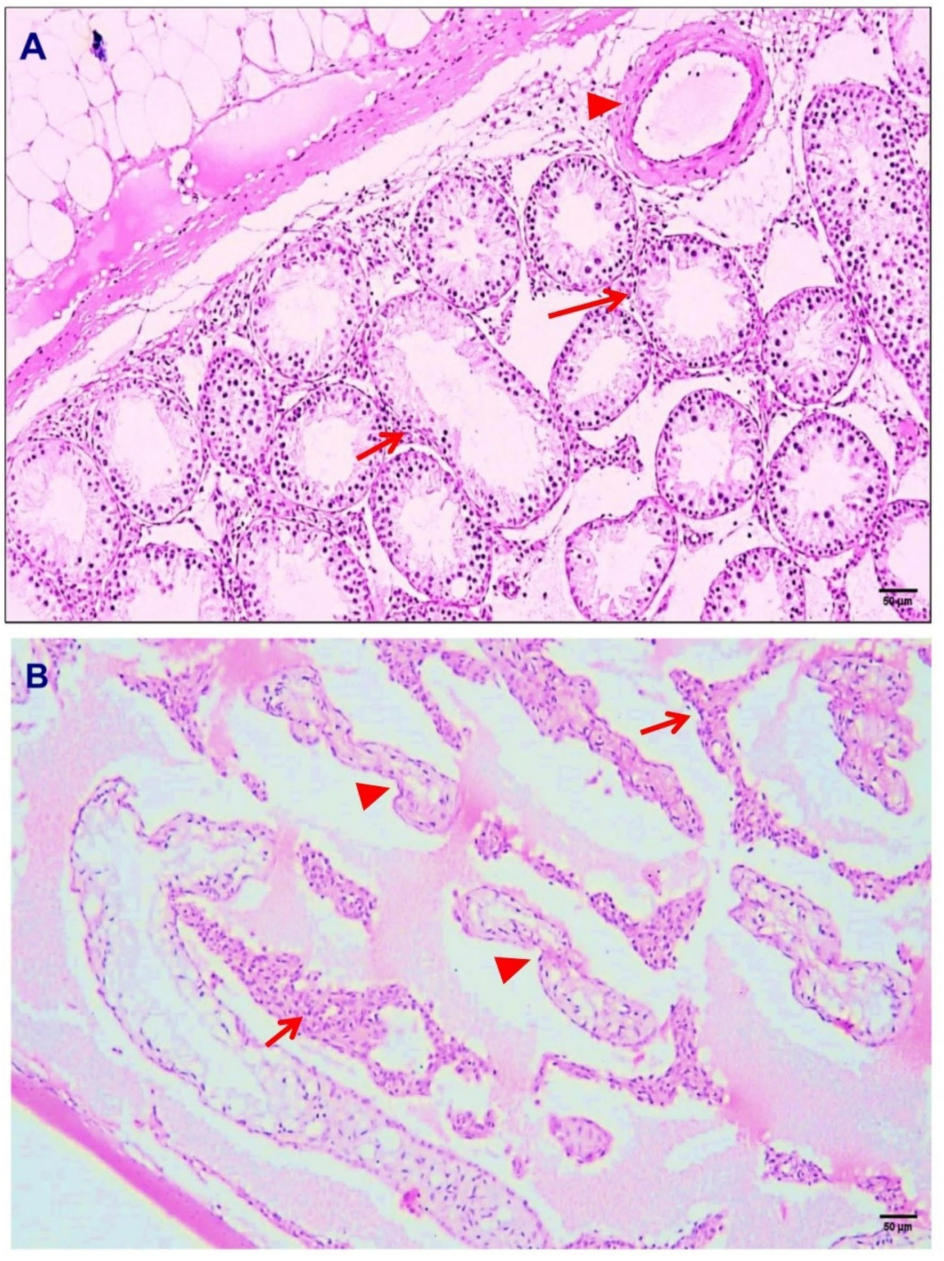
Sections from the testes of Toxoplasma-infected rats (H&E, ×100) showing (**A**) hypospermatogenic seminiferous tubules (arrow) with edema, mononuclear cell infiltration, fibrosis and thick walled blood vessels (arrowhead) and (**B**) Leydig cell hyperplasia (arrow) in addition to degenerated atrophied seminiferous tubules (arrowhead).

In vacuolar degeneration, the cell displayed small vacuoles, which represented distended and pinched segments of the endoplasmic reticulum. Injured cells also showed increased eosinophilic staining of the cytoplasm, which became more pronounced with progression to necrosis (Fig. 1B). Moreover, *Toxoplasma* cysts were also observed in seminiferous tubules (Fig. 1C). Additional observations included seminiferous tubule lumens that were comparatively broad and sparsely or completely devoid of sperm. Spermatocytes and exfoliated spermatogonia were observed in the lumen. Dystrophic calcification is detected focally in examined testicular tissue (Fig. 1D, E, F). Because of congestion and edema in the interstitial intertubular connective tissue, the tubular cross sections were broadly spaced in the interstitial intertubular tissue. Thick walled congested blood vessels, mononuclear cell infiltration and Leydig cell hyperplasia were observed in the interstitial tissues (Fig. 2).

**Fig. 3:**
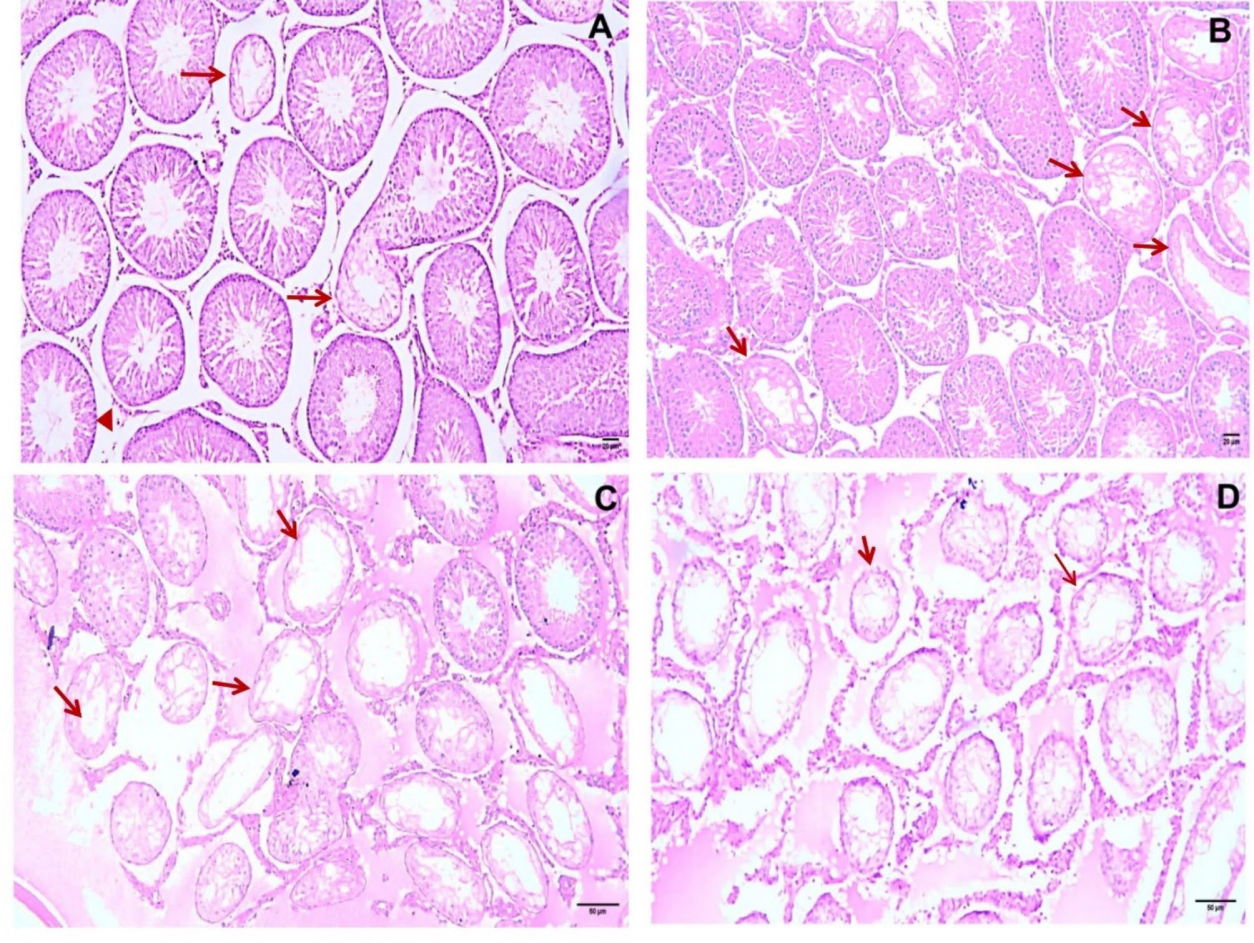
Sections from the testes of rats infected with *T. gondii* showing grades of seminiferous tubule degeneration (H&E, ×200): (**A**) minimal (Grade I) and hypospermatogenic seminiferous tubules (arrowhead); (**B**) mild grade II; (**C**) moderate grade III; (**D**) severe grade IV.

During the observation period, the Sertoli cell number in the tubular sections (Index 20 A) increased gradually according to the metric analysis, attaining greater significance by the twelfth week postinfection. Additionally, in contrast to the control group, there was a constant significant decrease in the number of spermatids (Index 20B) throughout the observation period. The minor diameters of the seminiferous tubules (Index 50) significantly increased during the eleventh and twelfth weeks after infection. Conversely, during the observation period, there was a progressive decrease in the number of primary leptotene spermatocytes (Index 250), which was most noticeable between weeks 10 and 12 after infection compared to that in the control group. Testicular sections from rats infected with *Toxoplasma* had varying degrees of seminiferous tubule degeneration, which was identified based on the lack of sperm and degeneration of spermatogenic cells (Table 2) (Fig. 3).

**Table 2:** Grading of seminiferous tubule degeneration (Index 200) in *Toxo*plasma-infected rats via necropsy.

Duration of infection (week)	*n*. of rats showing Toxoplasma cysts	Group median degeneration grade
7	15	1.0
8	15	1.0
9	15	2.0 ^ab^
10	15	3.0 ^ab^
11	15	3.0 ^abc^
12	15	4.0 ^abc^
Significance**		z = 58.57
		p = < 0.001

n: number.

Grading of seminiferous tubule degeneration according to the percentage of degenerating tubules among 200 randomly selected seminiferous tubules.

**: Kruskal‒Wallis test and Dunnet’s post hoc test (significant if *p* < .05).

a: Significant vs. 7th week, b: significant vs. 8th week, c: significant vs. 9th week.

### Assessment of the degree of infection

**Fig. 4:**
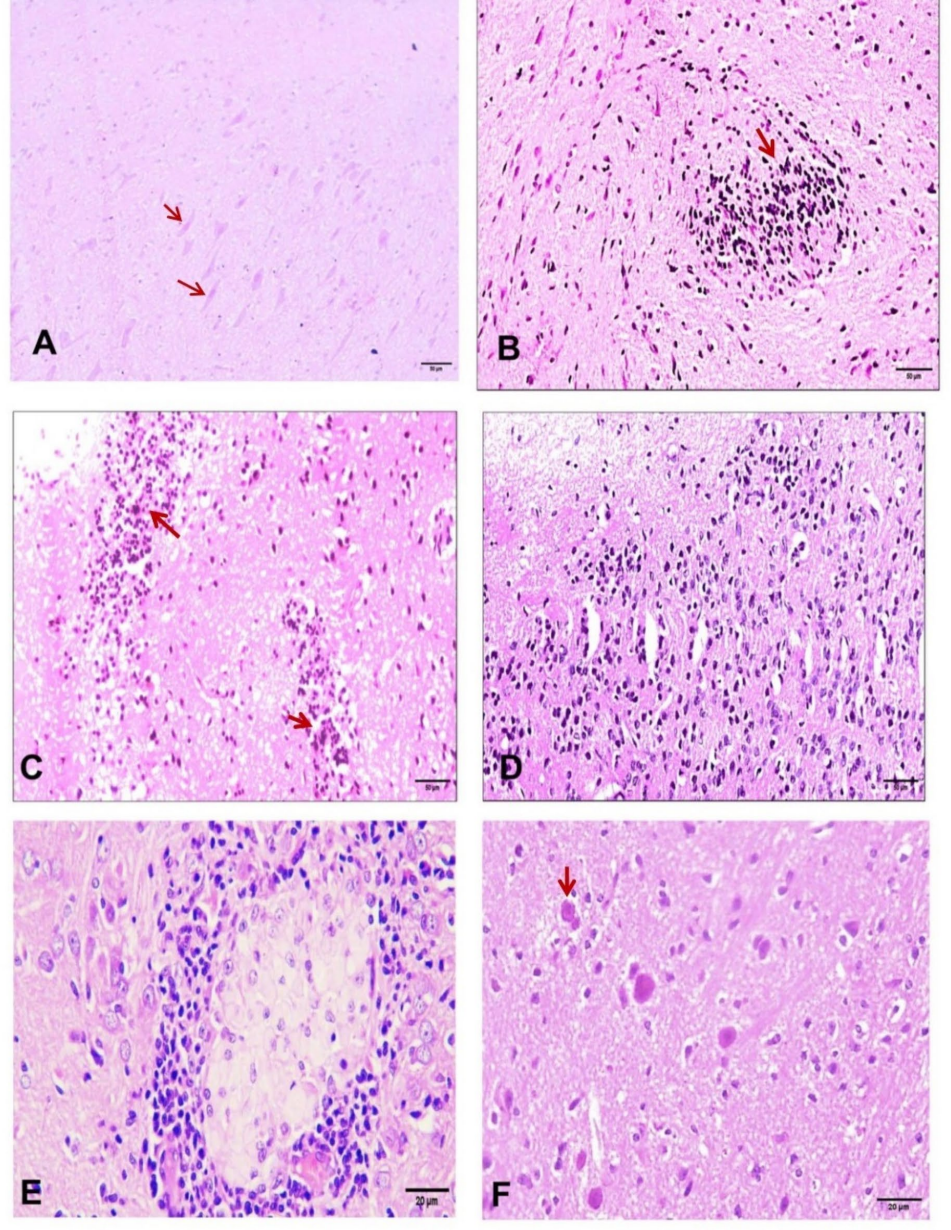
Histological findings of the rat brain (**A**) Section from the brain of a control rat demonstrating normal neurons (arrow) and brain architecture (H&E, ×200); (**B**) Section from the brain of a Toxoplasma-infected rat showing single minimal lesion (score I) with mononuclear cell infiltration (arrow) (H&E, ×200); (**C**) Multifocal minimal (score II) mononuclear cell infiltration (arrows) (H&E, ×200); (**D**) Single moderate (score III) mononuclear cell infiltration (H&E, ×200); (**E**) noncaseating epithelioid granuloma (score IV) (H&E, ×400); (**F**) Toxoplasma cyst (arrow) (H&E, ×400).

A gradual increase in the mean brain cyst count was observed throughout the observation period. However, no intergroup significance according to post hoc tests was detected (Table 3). Histopathological examination of brain sections from rats with *Toxoplasma* infection revealed various degrees of perivascular and interstitial inflammatory infiltration, neuronal damage, astrocytosis and meningitis. Post hoc testing clearly revealed a progressive, non significant increase in the median brain lesion grade that synchronized with infection progression, but the difference was not significant (Table 4; Fig. 4).

**Table 3:** Mean *Toxoplasma* brain cyst count in *rats infected with T. Gondii* at necropsy.

Infection duration in weeks	*n*. of rats necropitized	*n*. of rats showing Toxoplasma cysts^x^		Mean cyst count* (± SD)
**7**	17	15		4800.0 1 (± 526.44)
**8**	16	15		4806.67 (± 455.86)
**9**	15	15		4813.33 (± 516.67)
**10**	16	15		4820.0 1(± 522.63)
**11**	15	15		4833.33 (± 528.70)
**12**	16	15		4846.67 (± 539.66)
Significance**				**f = 0.017**
				*p* = 1.00

n: number, x: brain homogenate, *: number of cysts/ml brain homogenate x 2. **One-way ANOVA and Tukey’s post hoc test (significant if , P  < 0.05).

**Table 4:** Significance of brain lesion grade in *Toxoplasma-*infected rats.

Infection duration in weeks	*n*. of rats showing T.gondii cysts	Brain lesion grade* median (IQR)
**7**	15	1.0 (1.0–2.0)
**8**	15	1.0 (1.0–3.0)
**9**	15	2.0 (1.0–3.0)
**10**	15	2.0 (1.0–3.0)
**11**	15	2.0 (1.0–3.0)
**12**	15	3.0 (2.0–4.0)
Significance*		**z = 10.02**
		***P*** **= .075**

n: number, IQR: interquartile range, *: Kruskal‒Wallis test and Dunn’s post hoc test (significant if P < .05).

### Correlations between the degree of infection, body weight, and gonadosomatic index

**Fig. 5:**
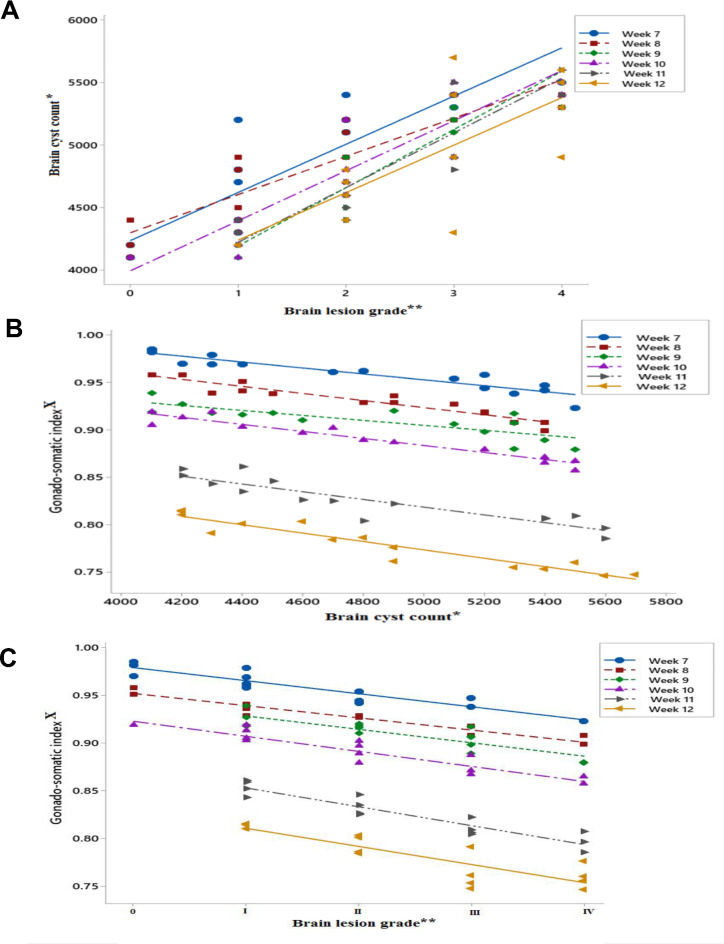
(**A**) Correlation between brain lesion grade and brain cyst count; (**B**) correlation between the GSI and brain cyst count; (**C**) correlation between the GSI and brain lesion grade. *: number of *T. gondii* cysts/ml brain homogenate X 2, **: According to Henry and Beverley^45^ and Tanaka et al.^44^, ×: Mean weight of both testes relative to 100 gm body weight at necropsy.

Brain lesion grading and brain cyst counts were parallel to one another. Therefore, as the infection progressed, the progressive increase in the group median lesion grade was not statistically significant. This increase in brain cyst number was correlated with infection (Fig. 5A). The association between brain cyst count and absolute body weight was significant only at the 8th and 9th weeks. Additionally, the correlations between brain lesion grade and absolute body weight were significant only at the 8th week. For the gonadosomatic index, the correlation was reversed throughout the observation period. An increase in brain lesion grade and brain cyst count was associated with a decrease in the gonadosomatic index (Fig. 5B&C). For the correlations between brain lesion grading, brain cyst counting, and metric analysis of the testes, an increase in the degree of infection was associated with a parallel increase in the effects of *Toxoplasma* infection on the spermatogenic parameters proven in this study by post hoc testing. There was a statistically significant **c**orrelation between the severity of seminiferous tubule degeneration, brain lesion grade and brain cyst count in *Toxoplasma-*infected rats at necropsy (see Supplementary Table S2 and Table S2 online).

## Discussion

Approximately one-third of people globally are infected with *T. gondii*. This protozoan has been detected in the semen and reproductive organs of both human and animal males. The effects of toxoplasmosis on sperm motility, count, and morphology were confirmed in rats. Our research investigated the influence of *T. gondii* infection on spermatogenesis by performing a testicular histomorphometric analysis of Wistar rats. In addition, these changes correlated with the degree of brain infection.

Compared to those of control naive rats, this study showed a slow but substantial decrease in the absolute body weight and the relative and absolute testes weights of the rats infected with *T. gondii*. Our findings regarding weight loss in *Toxoplasma-*infected rats are consistent with those of^46] and [47^, who reported a decrease in testicular weight. However, Tyebji et al.^13^ and Terpsidis et al.^48^ found no variation in the absolute weight of the testes in *T. gondii-*infected rats. It is more plausible that toxic shock-like syndrome, cytokine release and the host immunological response—possibly caused by an excess of TNFα—coincide with the body weight loss observed in the present study^49,50^. There are additional parasitic protozoa, such as *Trypanosoma cruzi*^51^, *Leishmania* species^52^, and *Plasmodium* species^53^, that can cause body weight loss due to parasitic diseases. Additionally, Al-Ghezy et al.^47^ reported a decline in the fetal body weight concurrently with *Toxoplasma* infection. Also, Dvorakova-Hortova et al.^42^, reported a decline in body weight concurrently with *Toxoplasma* infection.

The results of this study demonstrate that *T. gondii* infection negatively affects seminiferous tubules due to the small number of seminiferous tubules per optical field, a reduction in primary leptotene spermatocytes and spermatids, and an increase in the narrow diameters of the seminiferous tubules. Additional histopathological findings included *Toxoplasma* cysts, cellular interstitial infiltration, thinning of the spermatogenic cell rows, irregularly shaped Sertoli cell nuclei, wide gaps between cross sections of the seminiferous tubules, and occasional necrosis in the peri-tubular tissues.

Our findings are in accordance with those of Dvorakova-Hortova et al.^42^, who observed a decrease in leptotene spermatocytes and spermatids along with an increase in the narrow widths of the seminiferous tubules and an increase in the number of Sertoli cells in *Toxoplasma-*infected rats. The failure of energy-dependent ion pumps in the plasma membrane induced fluid accumulation, which resulted in vacuolation of the seminiferous epithelium. This failure to maintain ionic and fluid homeostasis leads to cell and endoplasmic reticulum dilatation^54^. Antimitotic agents, cytokine inhibitors, hypoxia, and androgen hypoxia are some of the variables that might lead to germ cell death. Abnormalities in the function of Sertoli cells could also cause this^55^.

Several forms of testicular lesions were detected in rats infected with *Toxoplasma*. Several studies have shown increased rates of spermatogenic cell apoptosis^56] and [56^. Additionally, Arantes et al.^14^ observed hydropic degeneration and fibrosis in dogs. Lopes et al.^57^ observed interstitial multifocal infiltration, diffuse testicular degeneration, and focal calcification in the testes of rats infected with *T. gondii*. Using immunohistochemistry, *Toxoplasma* parasites were found in the lesions of the rats. Jassem^58^ observed Leydig cell degeneration, multinucleated giant cell infiltration, and seminiferous tubule collapse. Rats infected with *Toxoplasma* were found to have *Toxoplasma* cysts in their testes and epididymis by^13,47,57^.

For somatic and male germ cells to proliferate, differentiate, and mature, they need to communicate, and this communication is regulated at several levels, including hormonal and epigenetic levels. Disruption of this fragile mechanism will increase the likelihood of developing abnormal phenotypes in the morphology of the testes or reproductive status^42^.

In this study, *Toxoplasma* cysts were found in the testes of rats. This finding coincides with that of Haskell et al.^21^, who reported *T. gondii* tachyzoites in the testes of males infected with human immune deficiency virus, whereas Wong et al.^59^ reported the presence of cysts harboring bradyzoites in the testes of immunocompetent individuals.

The mean diameter of a seminiferous tubule correlates directly with the number of Sertoli cells, as these cells are the key constructing component of the seminiferous tubule^60^; therefore, it was anticipated that synchronous with the increase in the Sertoli cell number, there would be a decrease in the narrow diameters of the seminiferous tubules as a result of an expected thickening of the seminiferous tubule epithelial linings, but the reverse would occur. The only explanation for this finding is that although Sertoli cells exhibit compensatory hyperplasia in response to a decrease in the number of germ cells, the rate of delay in the recruitment of germ cells in the spermatogenic cycle and/or the rate of their apoptosis greatly exceeds the rate of proliferation of Sertoli cells such that the diameter of the narrow seminiferous tubules increases and does not decrease.

Our findings indicated that there is a statistically significant correlation between the impact of latent toxoplasmosis and the degree of infection indicated by the number of *Toxoplasma* brain cysts and the degree of toxoplasmosis lesions in the brain. *Toxoplasma* cysts were detected in both rat brain hemispheres. Although *T. gondii* most frequently infects the basal ganglia and corticomedullary junction, postmortem brain examination reveals general involvement of both hemispheres^61^. Chronic *T. gondii* infection results in persistent inflammation and disruption of the blood–brain barrier, which facilitates the entry of circulating cytokines into brain tissue^62^. Moreover, cytokines such as tumor necrosis factor, interleukin-6, IL-12b, and interferon-gamma are thought to be markers for *T. gondii* infection in the mouse brain^63^ and are positively correlated with cyst burden^64^.

## Conclusion

Our study revealed that latent toxoplasmosis adversely affects the metrics and normal histology of male rat testes. A substantial correlation exists between the degree of *T. gondii* infection and this detrimental effect. Our findings suggest that future research on the impact of latent toxoplasmosis on male reproductive functions should be prioritized.

## Electronic supplementary material

Below is the link to the electronic supplementary material.

Supplementary Material 1

Supplementary Material 2

## Data Availability

The datasets used and/or analysed during the current study are available from the corresponding author (NE) on reasonable request.

## Abbreviations

T.gondii: Toxoplasma gondii
H&E: hematoxylin and eosin
PBS: Phosphate Buffer Solution
GSI: gonadosomatic index
SPSS: Statistical Package for the Social Sciences
