# Supplementary material for: The degree of toxoplasmosis and testicular histomorphometry in rats
Source: Sci Rep. 2024 Nov 13;14:27896. doi: 10.1038/s41598-024-78653-3 (PMC11561302; doi:10.1038/s41598-024-78653-3)
Supplement: Supplementary file 1 — Supplementary Material 1 [file 41598_2024_78653_MOESM1_ESM.docx]

**Supplementary Table S1: Correlation between Indices 20A and 20B, brain cyst count and brain lesion grade in *Toxoplasma*-infected rats at necropsy**

| **R5** | | **Group lesion grade^4^** | **R3** | | **Cyst**  **count^2^** | **Index**  **20 B^1*^** | **R^5^** | | **Group lesion grade^4^** | **R^3^** | | **Cyst**  **count^2^** | **Index**  **20 A^1^** | **Duration of infection (week)** |
| --- | --- | --- | --- | --- | --- | --- | --- | --- | --- | --- | --- | --- | --- | --- |
| **P** | **r** |  | **P** | **r** |  |  | **P** | **r** |  | **P** | **r** |  |  |  |
| ˂0.001 | -0.971 | 1.0 | ˂0.001 | -0.971 | 4800.0  ±526.44 | 98.10  ±10.61 | <0.001 | 0.960 | 1.0 | <0.001 | 0.971 | 4800.0  ±526.44 | 15.0  ±1.36 | **7** |
| ˂0.001 | -0.978 | 1.0 | ˂0.001 | -0.972 | 4806.67  ±455.86 | 95.49  ±10.63 | ˂0.001 | 0.966 | 1.0 | ˂0.001 | 0.982 | 4806.67  ±455.86 | 15.03  ±1.38 | **8** |
| ˂0.001 | -0.945 | 2.0 | ˂0.001 | -0.945 | 4813.33  ±516.67 | 90.62  ±11.92 | ˂0.001 | 0.973 | 2.0 | ˂0.001 | 0.972 | 4813.33  ±516.67 | 15.27  ±1.36 | **9** |
| ˂0.001 | -0.977 | 2.0 | ˂0.001 | -0.925 | 4820.0  ±522.63 | 84.06  ±11.24 | ˂0.001 | 0.957 | 2.0 | ˂0.001 | 0.959 | 4820.0  ±522.63 | 15.61  ±1.09 | **10** |
| ˂0.001 | -0.987 | 2.0 | ˂0.001 | -0.914 | 4833.33  ±528.70 | 74.98  ±7.70 | ˂0.001 | 0.966 | 2.0 | ˂0.001 | 0.980 | 4833.33  ±528.70 | 16.05  ±1.11 | **11** |
| 0.001 | -0.640 | 3.0 | 0.001 | -0.640 | 4846.67  ±539.66 | 71.53  ±6.70 | <0.001 | 0.933 | 3.0 | <0.001 | 0.975 | 4846.67  ±539.66 | 16.22  ±1.07 | **12** |

: Index 20 A is the number of Sertoli cells per 20 seminiferous tubules cross sections calculated as the mean (±SD)

1*: Index 20 B is the number of spherical spermatids per 20 seminiferous tubules cross sections calculated as the mean (±SD), 2: Number of cysts/ ml brain homogenate X 2 (As the mean ±SD), 3: Correlation coefficient (Spearman's test) (1 vs. 2), 4: As the median, 5: Correlation coefficient (Spearman's test) (1 vs.4)

**Supplementary Table S2: Correlations between the indices 50 and 250, brain cyst count and brain lesion grade in *Toxoplasma*-infected rats at necropsy**

| **R5** | | **Group lesion grade^4^** | R3 | | **Cyst**  **count^2^** | **Index**  **250^1*^** | **R^5^** | | **Group lesion grade^4^** | **R^3^** | | **Cyst**  **count^2^** | **Index**  **50^1^** | **Duration of infection (week)** |
| --- | --- | --- | --- | --- | --- | --- | --- | --- | --- | --- | --- | --- | --- | --- |
| P | r |  | P | r |  |  | P | **R** |  | P | **r** |  |  |  |
| ˂.001 | -0.960 | 1.0 | ˂.001 | -0.971 | 4800.0  ±526.44 | 686.73  ±74.25 | ˂0.001 | 0.943 | 1.0  (0.0-4.0) | ˂0.001 | 0.977 | 4800.0  ±526.44 | 181.8  ±13.64 | **7** |
| ˂.001 | -0.969 | 1.0 | ˂.001 | -0.978 | 4806.67  ±455.86 | 668.40  ±74.38 | ˂0.001 | 0.966 | 1.0  (0.0-4.0) | ˂0.001 | 0.969 | 4806.67  ±455.86 | 184.27  ±15.22 | **8** |
| ˂.001 | -0.929 | 2.0 | ˂.001 | -0.945 | 4813.33  ±516.67 | 634.4  ±83.41 | ˂0.001 | 0.974 | 2.0  (0.0-4.0) | ˂0.001 | 0.971 | 4813.33  ±516.67 | 190.47  ±15.26 | **9** |
| ˂.001 | -0.968 | 2.0 | ˂.001 | -0.977 | 4820.0  ±522.63 | 588.40  ±78.66 | ˂0.001 | 0.952 | 2.0  (0.0-4.0) | ˂0.001 | 0.960 | 4820.0  ±522.63 | 193.07  ±17.51 | **10** |
| ˂.001 | -0.949 | 2.0 | ˂.001 | -0.987 | 4833.33  ±528.70 | 524.87  ±53.93 | ˂0.001 | 0.965 | 2.0  (1.0-4.0) | ˂0.001 | 0.982 | 4833.33  ±528.70 | 201.47  ±14.35 | **11** |
| ˂.001 | -0.949 | 3.0 | ˂.001 | -0.970 | 4846.67  ±539.66 | 490.07  ±50.79 | ˂0.001 | 0.932 | 3.0  (1.0-4.0) | ˂0.001 | 0.978 | 4846.67  ±539.66 | 206.8  ±16.67 | **12** |

1: Index 50 is the minor diameters of 50 seminiferous tubules calculated as the mean (±SD), 1*: Index 250 is the number of primary leptotene spermatocytes per 250 Sertoli cells calculated as the mean (±SD), 2: Number of cysts/ ml brain homogenate X 2 (As the mean ±SD), 3: Correlation coefficient (Spearman's test) (1 vs. 2), 4: As the median, 5: Correlation coefficient (Spearman's test) (1 vs. 4)
