# Supplementary material for: The degree of toxoplasmosis and testicular histomorphometry in rats
Source: Sci Rep. 2024 Nov 13;14:27896. doi: 10.1038/s41598-024-78653-3 (PMC11561302; doi:10.1038/s41598-024-78653-3)
Supplement: Supplementary file 2 — Supplementary Material 2 [file 41598_2024_78653_MOESM2_ESM.docx]

**Figure Legends:**

**Fig. 1**. Histological analysis of the testes of the rats (H&E, ×200). (A) A section of a control rat testis demonstrating the normal structure of the seminiferous tubules; Arrow head: normal seminiferous tubule full with spermatogenic cells and sperm; LC: Leydig cells; S indicates spermatozoa; Section from the testis of *Toxoplasma*-infected rat showing (B) vacuolar degeneration (arrow) and necrosis of spermatogenic cells (H&E, ×200); (C) *Toxoplasma* cyst (arrow), degeneration of spermatogenic cells with apoptotic changes (arrow head) and absent spermatids (H&E, ×400); SC: Sertoli cells; (D) detached germ cells (arrow) in the lumen of degenerated seminiferous tubules (H&E, ×200); (E) irregularly outlined seminiferous tubules with degenerated spermatogenic cells, detached germ cells (arrow) in the lumen and no sperm (arrow head) (H&E, ×200); (F) degenerated seminiferous tubules with absence of germ cells (arrow) and dystrophic calcification (arrow heads) (H&E, ×200).

**Fig. 2.** Sections from the testes of *Toxoplasma*-infected rats (H&E, ×100) showing (A) hypospermatogenic seminiferous tubules (arrow) with edema, mononuclear cell infiltration, fibrosis and thick walled blood vessels (arrowhead) and (B) Leydig cell hyperplasia (arrow) in addition to degenerated atrophied seminiferous tubules (arrowhead).

**Fig. 3.** Sections from the testes of rats infected with *T. gondii* showing grades of seminiferous tubule degeneration (H&E, ×200): (A) minimal (Grade I) Seminiferous tubules degeneration (arrow) and hypospermatogenic seminiferous tubules (arrowhead); (B) mild grade II seminiferous tubules degeneration (arrow); (C) moderate grade III seminiferous tubules degeneration (arrow); (D) severe grade IV seminiferous tubules degeneration (arrow).

**Fig. 4.** Histological findings of the rat brain (A) Section from the brain of a control rat demonstrating normal neurons (arrow) and brain architecture (H&E, ×200); (B) Section from the brain of a *Toxoplasma*-infected rat showing single minimal lesion (score I) with mononuclear cell infiltration (arrow) (H&E, ×200); (C) Multifocal minimal (score II) mononuclear cell infiltration (arrows) (H&E, ×200); (D) Single moderate (score III) mononuclear cell infiltration (H&E, ×200); (E) noncaseating epithelioid granuloma (score IV) (H&E, ×400); (F) Toxoplasma cyst (arrow) (H&E, ×400).

**Fig. 5.**  (A) Correlation between brain lesion grade and brain cyst count; (B) correlation between the GSI and brain cyst count; (C) correlation between the GSI and brain lesion grade. *: number of *T. gondii* cysts/ml brain homogenate X2, **: According to Henry and Beverley (1969) and Tanaka et al. (2013), ×: Mean weight of both testes relative to 100 gm body weight at necropsy.
